# Crack Length Measurement Using Convolutional Neural Networks and Image Processing

**DOI:** 10.3390/s21175894

**Published:** 2021-09-01

**Authors:** Yingtao Yuan, Zhendong Ge, Xin Su, Xiang Guo, Tao Suo, Yan Liu, Qifeng Yu

**Affiliations:** 1 School of Aeronautics, Northwestern Polytechnical University, Xi’an 710072, China; yuanyingtao@mail.nwpu.edu.cn (Y.Y.); 13817583533@163.com (Z.G.); suxin.ds@mail.nwpu.edu.cn (X.S.); suotao@nwpu.edu.cn (T.S.); yuqifeng58@139.com (Q.Y.); 2 International Research Laboratory of Impact Dynamics and its Engineering Application, Xi’an 710072, China; 3 Institute of Intelligent Optical Measurement and Detection, Shenzhen University, Shenzhen 518060, China; yan_liu@mail.nwpu.edu.cn

**Keywords:** crack length, image processing, convolutional neural network, fatigue crack detection

## Abstract

Fatigue failure is a significant problem in the structural safety of engineering structures. Human inspection is the most widely used approach for fatigue failure detection, which is time consuming and subjective. Traditional vision-based methods are insufficient in distinguishing cracks from noises and detecting crack tips. In this paper, a new framework based on convolutional neural networks (CNN) and digital image processing is proposed to monitor crack propagation length. Convolutional neural networks were first applied to robustly detect the location of cracks with the interference of scratch and edges. Then, a crack tip-detection algorithm was established to accurately locate the crack tip and was used to calculate the length of the crack. The effectiveness and precision of the proposed approach were validated through conducting fatigue experiments. The results demonstrated that the proposed approach could robustly identify a fatigue crack surrounded by crack-like noises and locate the crack tip accurately. Furthermore, crack length could be measured with submillimeter accuracy.

## 1. Introduction

Fatigue cracks caused by repetitive loads, which are of great concern for structural safety, always exist in old structures including airplane and highway bridges [1]. Fatigue crack propagation testing is an essential method of studying metallic or structural fatigue life prediction in fracture mechanics. Crack length is one of the most relevant parameters that needs to be recorded during laboratorial tests. 

In recent decades, extensive research on monitoring crack length propagation has been carried out. Existing approaches including the human inspection method, the electrical method [2], the compliance method [3], and acoustic emission technology [4] all have limitations for application during a fatigue test. The human inspection method requires large testing times since it needs to interrupt the fatigue test to manually measure crack lengths. The accuracy of the electrical method is easily affected by the experiment conditions and environment. Complicated calibration is needed when applying the compliance method. Acoustic emission technology is a new nondestructive testing method. It records and analyzes the signals released by materials or structures when deformation or damage occurs to detect the damage location and predict the time to failure. However, this technology relies on a good correlation between acoustic emission data and the damage mechanism, while the experimental results will be affected by environmental noise [5]. Therefore, the application of acoustic emission technology for monitoring the length of cracks is limited. 

Vision-based crack detection methods have been widely studied over recent decades for their advantages of non-contact, high precision, and good real-time performance [6]. Many methods have been established based on image processing techniques including edge detection [7], Hough transform [8], image segmentation [9], identification and detection of feature points [10,11], the digital image correlation (DIC) method [12,13], and photogrammetry [14]. A general limitation of these approaches, however, is that most of these vision-based methods detect cracks by searching over the entire area of an image. As a result, there are difficulties in distinguishing true cracks from crack-like noises such as scratches and structure boundaries [15]. At the same time, accurate detection of crack tips, which is key for crack length measurement, remains challenging [10].

In recent years, machine learning and convolutional neural networks have shown a strong capability for feature extraction and target detection, and they have been used in structural health monitoring (SHM). For example, Dong [16] established a CNN model to identify microseismic events and blasts accurately. Yu [17] put forward a DCNN-based method to localize damages to smart building structures with high accuracy on raw noisy signals. Many researchers have carried out studies for crack detection based on CNN. Cha [18] used trained CNN and sliding window techniques to detect cracks from images. Other researchers [19,20] used trained CNN to divide every crack pixel from the background in the image. Although these approaches can detect the cracks with high reliability even surrounded by crack-like noises, it would be difficult to accomplish quantitative detection of crack length. 

Previously, the authors investigated a crack propagation detection method [21] using classification-based CNN and an improved canny edge-detection algorithm, and verified the accuracy and efficiency of the method via central hole specimens. However, the classification-based CNN and the edge-detection algorithm only used part of the crack structure information, which reduced the reliability of the method. 

In this study, we proposed an improved crack length measurement framework based on detection-based CNN and digital image processing. This framework combined the excellent feature extraction capabilities of detection-based CNN with the quantitative detection capabilities of traditional vision-based methods. This CNN-based model was established to robustly detect whole true cracks surrounded by crack-like noises. A new image-processing algorithm was proposed to accurately locate the crack tip, which could be used to calculate the length of the crack. 

The manuscript is organized as follows. Section 2 presents the synopsis of the proposed algorithm first, then the architecture of CNN used in this paper and the methodology of crack tip detection are introduced and thoroughly detailed. Section 3 illustrates the experimental process and results including training dataset preparation, CNN training results, crack area location results, crack tip detection results, and crack length measurement results. Section 4 concludes the paper. 

## 2. Methodology

### 2.1. Overview of the Proposed Method

**Figure 1:**
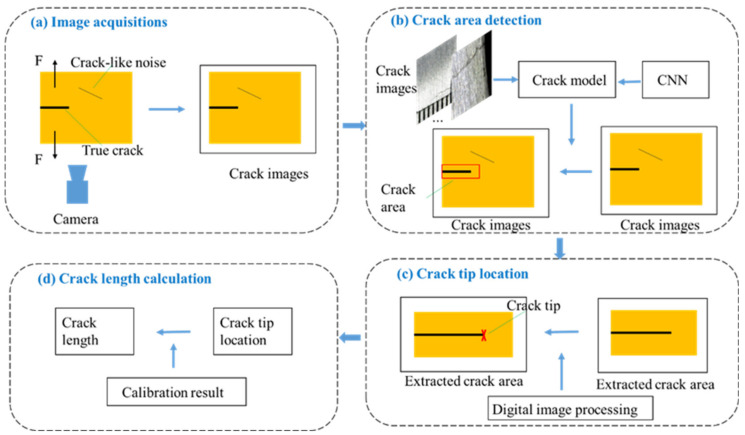
The scheme of the crack length measurement method: (**a**) image acquisitions; (**b**) crack area detection; (**c**) crack tip location; (**d**) crack length calculation.

The crack length measurement method proposed in this paper is shown in Figure 1, with more technical details described in the rest of this section. To begin, a fixed camera was applied to acquire images while a specimen was subject to a fatigue crack under a fatigue load F, as Figure 1a shows. These crack images contained not only crack information but also crack-like noises. At the same time, the experimental information such as fatigue cycles could be recorded. Then, to achieve robust crack detection, a convolutional neural network trained by labeled crack images was used to detect the region of the crack with the interference of a non-crack edge, as shown in Figure 1b. The architecture of the applied CNN is introduced in Section 2.2, and the process of training dataset preparation and the crack area detection results are detailed in Section 3.1 and Section 3.2. Next, a crack tip location method based on digital image processing was used to detect the crack tip from the extracted crack area, as shown in Figure 1c. The principle of the proposed method is illustrated in Section 2.3, and the experimental results are presented in Section 3.3. In the end, the crack length was calculated with the calibration result, as shown in Figure 1d. The introduction of a validation experiment and the results analysis are described in Section 3.4. 

### 2.2. Crack Area Location Method 

**Figure 2:**
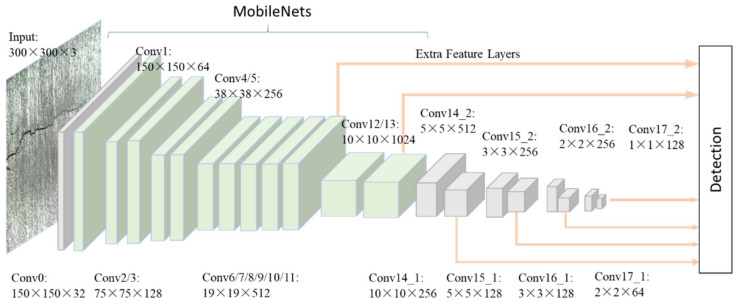
The architecture of the CNN for crack area location.

This section explains the overall architecture of CNN used in this study for crack detection. CNN is a robust method for detecting different objects and has been widely used in automatic drive and intelligent housing systems. CNN extracted the features of objects by convolution layers which could be recognized as the filters. Then, feature maps that indicated the locations and strength of the input object were calculated. In the end, the networks learned the best parameters by adjusting themselves to reduce the error on the dataset. Thus, CNN could be used to distinguish cracks from non-crack noises. Figure 2 shows the details of the CNN architecture. The input crack image was first resized into 300 × 300 × 3, where each dimension indicated height, width, and channel, respectively. Then, the crack image passed through the architecture with 6 feature maps extracted, including conv11, conv13_2, conv14_2, conv15_2, conv16_2, and conv17_2. The term “conv” refers to the convolution layer. These feature maps were used to train and detect the location of cracks. The meta-architecture of the CNN was a Single Shot Detector (SSD) [22], and the feature extractor was MobileNets [23]. 

**Figure 3:**
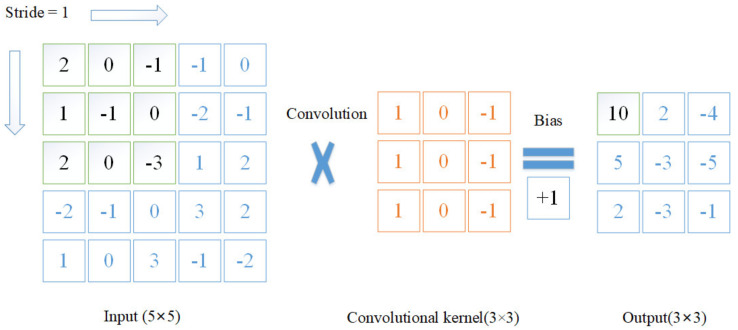
The principle of convolutional layers.

A convolutional layer is one of the most important parts in CNN. As shown in Figure 3, the size of the input data was 5 × 5, the size of the convolutional kernel was 3 × 3, and the size of the output was 3 × 3. The convolutional layer performed element-by-element multiplications. The multiplied values were summed and, adding bias, we derived the output value. The stride was a hyperparameter, describing how many of the kernel’s columns and rows slid at a time across the input data’s width and height. One of the advantages of convolution was that it could reduce input data size, and as a result the computational cost was reduced. The feature maps mentioned above are the results of data input after the convolution operation. However, the operation of convolution was time consuming, so we replaced it with a more efficient network. 

**Figure 4:**
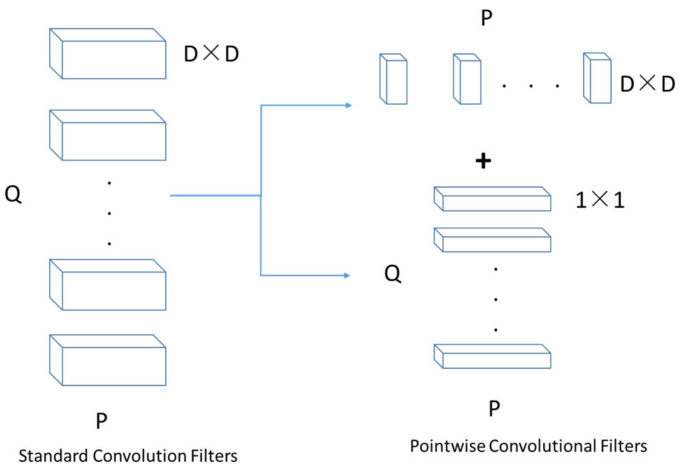
The principle of MobileNets.

MobileNets can effectively reduce the number of parameters in CNN architecture. The principle of MobileNets is shown in Figure 4. The standard convolutional layer was replaced by depthwise separable convolution, which factorized the standard convolution into two parts, a depthwise convolution and a 1 × 1 convolution (also called a pointwise convolution). If an N × N × P feature map was taken as the input by a standard convolution, and a D × D × Q feature map was produced, then the computational cost was N × N × P × D × D × Q for each layer. When depthwise separable convolution was applied, the computational cost of a depthwise convolution was P × N × N × D × D, and the computational cost of a pointwise convolution was P × Q × N × N × 1 × 1. The number of parameters reduced from P × Q × N × N × D × D to P × N × N × D × D+ P × Q × N × N × 1 × 1. As a result, the time cost for detecting cracks would be lower.

In this study, we used the Rectified Linear Unit (ReLU) [24] as the activation function for all layers. Briefly, the ReLU had no bounded outputs for its positive input values and the gradients are either zeros or ones. This enabled ReLU to compute faster and could achieve better accuracies. The function of ReLU is shown as:

(1) $$y=\left\{\begin{array}{l} x\begin{array}{cc} & if:x>0 \end{array}\\ 0\begin{array}{cc} & if:x<0 \end{array} \end{array}\right.$$

**Figure 5:**
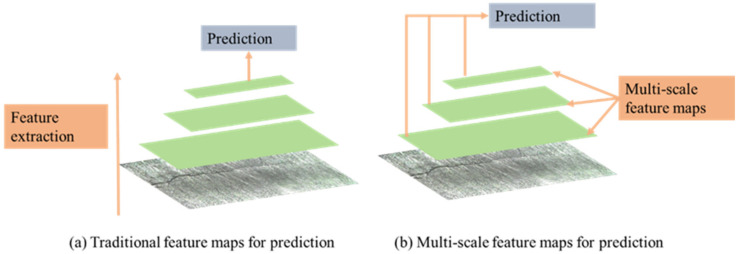
Multi-scale crack feature maps for detection.

**Figure 6:**
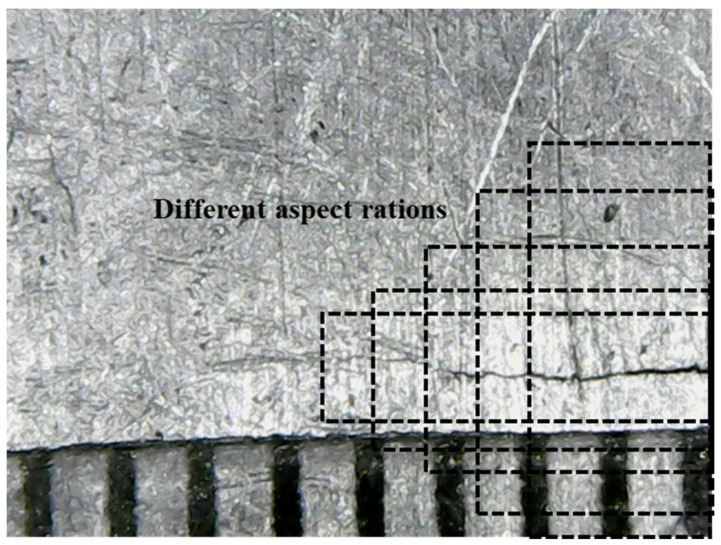
Different aspect ratios for crack detection.

The meta-architecture used in this study was SSD. This architecture could detect objects faster without losing accuracy [25], and provided the possibility to detect cracks in real time. It used different resolutions of feature maps to predict different length cracks. As shown in Figure 5, multi-scale feature maps were extracted using a set of convolutional layers. These maps decreased in size and made predictions of cracks at multiple scales. Large-scale feature maps could be used to predict small cracks and small-scale feature maps could be used to predict large cracks. Another method it used to detect different scale cracks was setting different aspect ratios for default bounding boxes. As shown in Figure 6, the crack was distributed in a linear fashion and a square area was not suitable for the prediction of cracks. Therefore, we set five different values including 1, 2, 3, 1/2, and 1/3 as aspect ratios for default boxes. Each feature map cell predicted the offsets relative to these default bounding boxes.

The overall loss function was a weighted sum of the localization loss and the confidence loss:

(2) $$L(x,c,l,g)=\frac{1}{N}(L_{conf}(x,c)+L_{loc}(x,l,g))$$

where *N* is the number of matched default boxes; $L_{conf}$ is the confidence loss; $L_{loc}$ is the localization loss; x={1,0} is an indicator for matching the i-th default box (l) to the j-th ground-truth box (g) parameters of the crack; c means the values of confidence.

The localization loss is a Smooth L1 loss [26,27]:

(3) $$\begin{array}{l} L_{loc}(x,l,g)=\sum_{i\in Pos}^{N}\sum_{m\in \{cx,cy,w,h\}}x_{ij}\cdot smooth_{L1}(l_{i}-\overset{\wedge }{g_{j}^{m}})\\ \overset{\wedge }{g_{j}^{cx}}=(g_{j}^{cx}-d_{i}^{cx})/d^{w};\overset{\wedge }{g_{j}^{cy}}=(g_{j}^{cy}-d_{i}^{cy})/d^{h}\\ \overset{\wedge }{g_{j}^{w}}=\log\left(\frac{g_{j}^{w}}{d_{i}^{w}}\right);\overset{\wedge }{g^{h}}=\log\left(\frac{g_{j}^{h}}{d_{i}^{h}}\right) \end{array}$$

where (cx,cy) means the center of the default bounding box (d), and w and h are the width and height of the default bounding box. 

The confidence loss refers to the reliability of predictions that the box contains a crack:

(4) $$L_{conf}(x,c)=-\sum_{i\in Pos}^{N}x_{ij}\cdot \log({\overset{\wedge }{c}}_{i}^{1})-\sum_{i\in Neg}\log({\overset{\wedge }{c}}_{i}^{0})$$

### 2.3. Crack Tip Detection Method

**Figure 7:**
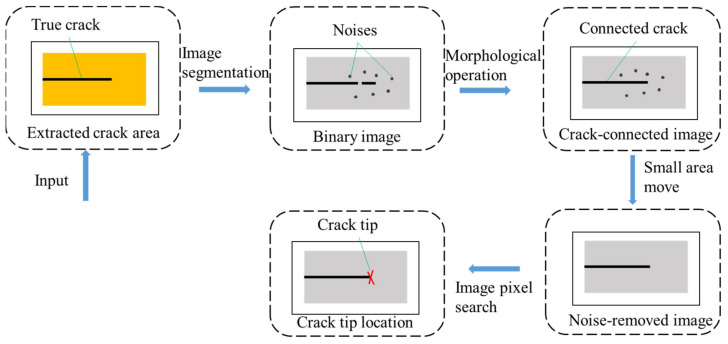
The method proposed to detect crack tips.

Once the crack area was detected using CNN, the next step was to robustly locate the crack tip. As shown in Figure 7, the algorithm proposed to locate the crack tip could be divided into four steps. First, an image threshold segmentation method was applied to divide the crack and the background. Then, we used an image morphological operation method to connect the broken crack. Next, a noise-free crack image was obtained after removing small-area noises. In the end, the crack tip could be located by searching the image. 

In this study, we used a global threshold segmentation method to divide the crack and background to keep the information about the crack tip. During the process of crack propagation, the brightness in the location of the crack was darker than the background. Therefore, we set a grayscale value *T* to separate the crack and the background. Every crack image pixel was divided into two groups after threshold segmentation. The function of threshold segmentation used in the study was:

(5) $$g(x,y)=\left\{\begin{array}{l} \begin{array}{cc} 0 & f(x,y)>T \end{array}\\ \begin{array}{cc} 255 & f(x,y)\leq T \end{array} \end{array}\right.$$

As the crack tip was small and would be easily disturbed by noises, the area of cracks, especially the crack tip, was disconnected after the operation of threshold segmentation. We needed to enhance the area of the crack before removing noises. 

**Figure 8:**
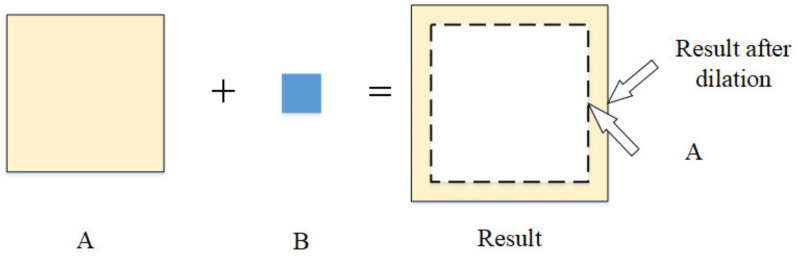
The principle of dilation.

**Figure 9:**
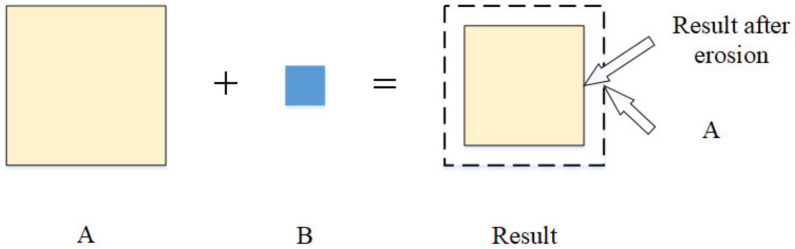
The principle of erosion.

Morphological operators [28] included dilation and erosion. The principle of dilation is shown in Figure 8. The dilation of region A by a structuring element B produced a new region, adding a layer of pixels to the boundary of regions. Dilation could fill in the holes and gaps between different regions. The principle of erosion is shown in Figure 9. The erosion of area A by a structuring element B produced a new region, which was smaller than A. Erosion could remove small-scale details, which meant it could be used to remove noises. The closing operation dilated an image and then eroded the dilated image. This was useful for filling small holes and gaps while preserving the shape and size of the objects. Thus, we could use the closing operation to connect the crack and keep the shape of the crack. According to the shape of crack, we could use a rectangular structuring element to close the crack image. 

After the crack connection, we could label the regions by searching the binary image and recording the number of pixels. We found that the noises had a small area. Therefore, we could remove the noises by removing small-area objects. As a result, a clear crack binary image was obtained. In the end, we could search the pixel location of 255 and define the rightmost pixel as the crack tip.

## 3. Experimental Results and Analysis

### 3.1. Training Data Preparation and Parameter Initialization

**Figure 10:**
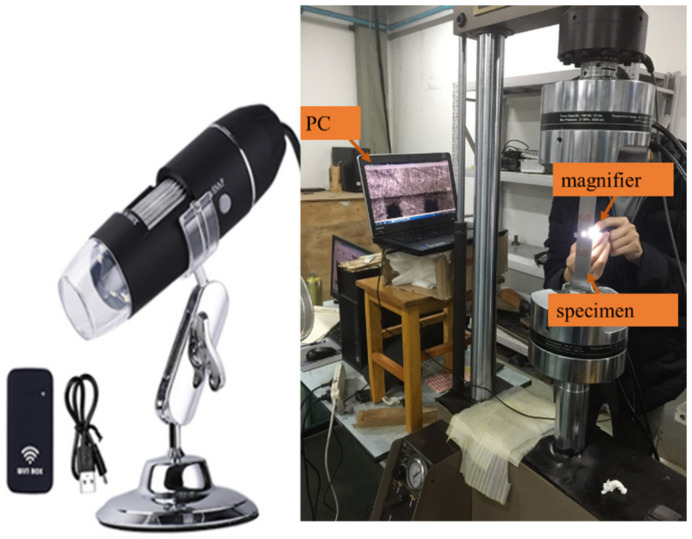
Experiments for collecting crack data: the left image shows the measuring magnifier used in the experiments, and the right image shows the experimental environment.

**Figure 11:**
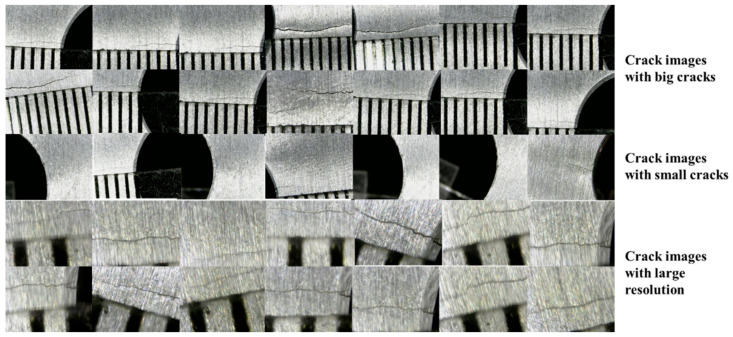
Examples of images used for training CNN.

CNN is a data-driven approach. Before training the CNN model, the authors carried out fatigue experiments to collect crack images. As shown in Figure 10, a measuring magnifier was used to capture the crack images. The total number of raw images was 4928 (all images with 640 × 480 pixel resolutions). Some examples of crack images used for training CNN are shown in Figure 11. There were three kinds of crack images including images with large cracks, images with small cracks, and crack images in larger resolution. 

These crack images were divided into two groups according to a fivefold cross-validation principle of 80% for training and 20% for validation. All the crack images were divided into two parts including 3928 images as training datasets and 1000 images as validation sets.

These crack images were manually labeled with LabelImg [29]. The information of the crack including the location and category in each image was recorded. In this study, the CNN model was built in the framework of TensorFlow. Before training the model, the dataset was converted into TFRecords Format, which is suitable for TensorFlow framework. At that point, we established a crack dataset. 

A crack was a new category for us to train and detect. The transfer learning method was not suitable for our CNN model, since research demonstrated that the performance relies on the similarity of two tasks [30,31]. We initialized the model parameters with a normal distribution with mean of 0 and standard deviation of 0.03, and the biases were initialized with a constant zero vector. According to Huang [25], the initial learning rate was set as 0.004. The batch size was set as 1 due to the limitation of our computer. 

### 3.2. CNN Training Results 

The training process was carried out on a workstation with a GPU (GeForce GTX 1080Ti). The code was programmed by Python 3.6, and the environment was established by TenorFlow1.12. The training process was performed in Windows systems. 

**Figure 12:**
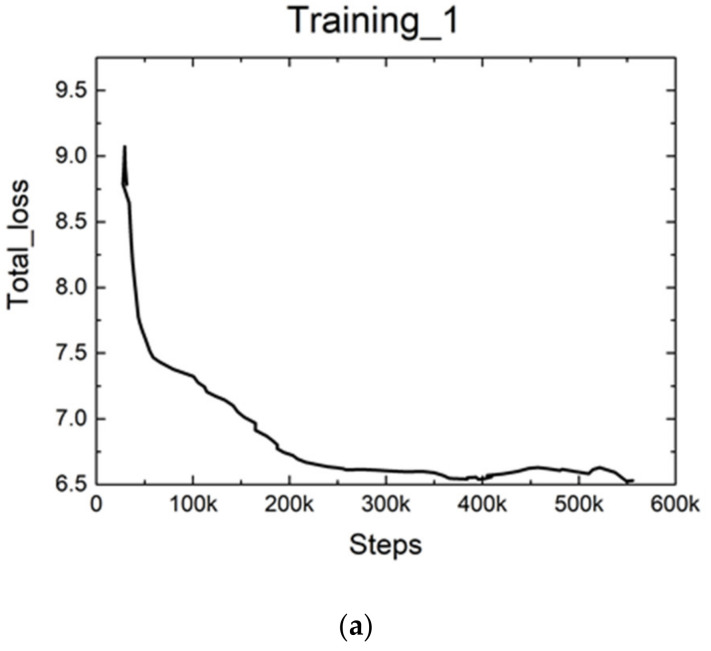

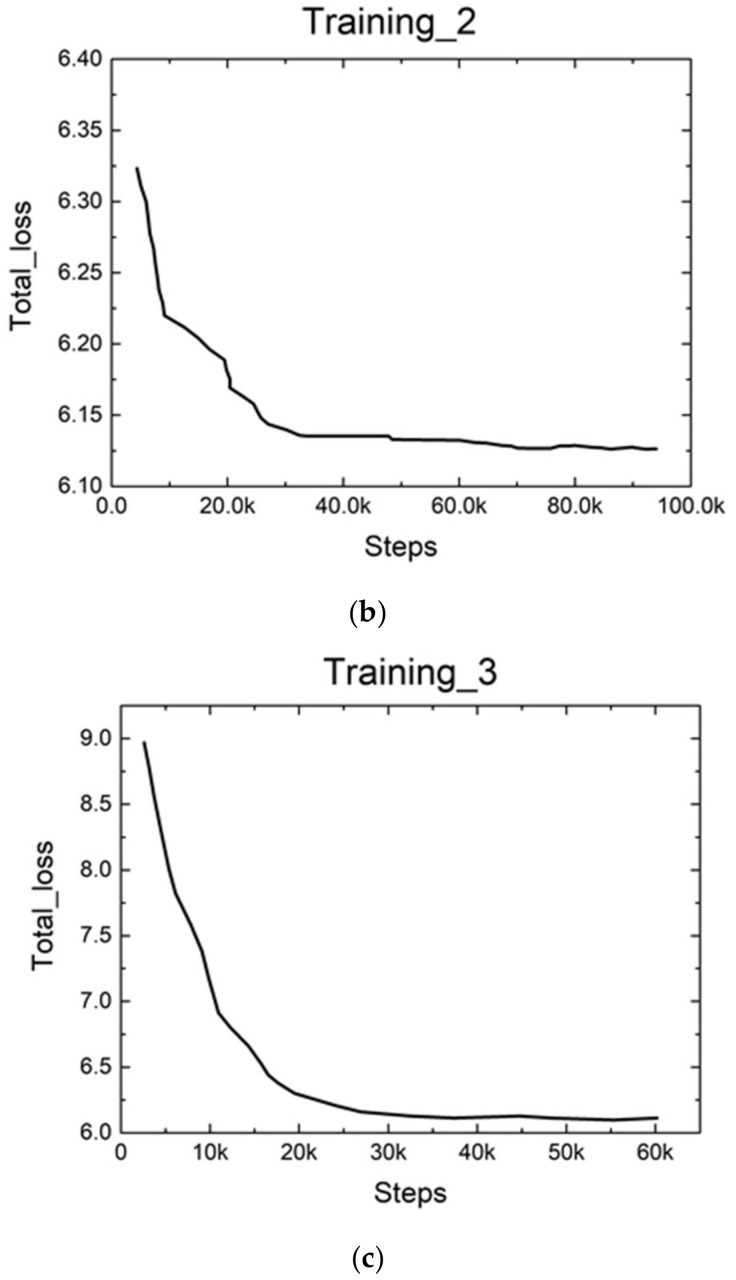
The total loss during the training process: (**a**) the total loss over the first training process; (**b**) the total loss over the second training process; (**c**) the total loss over the third training process.

The learning rate was manually adjusted over the training iterations, so that the parameters could converge to the global optimum. In general, larger learning rates were preferred in the beginning and smaller learning rates were used for fine-tuning. The total loss with the training process over steps is shown in Figure 12. In the first iteration, we set the learning rate equal to 0.004, and trained 55.6K steps. The value of total loss decreased quickly at the beginning and slowly stabilized at about 6.5 in the end. Then, we set the learning rate equal to 0.0004 to train 95K steps. The value of total loss stabilized at about 6.13 in the end. Finally, we used the learning rate of 0.00004 to train 60K steps. The value of total loss stabilized at about 6.1 in the end. 

**Figure 13:**
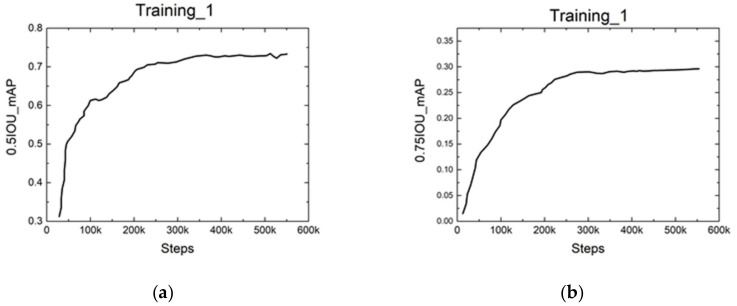

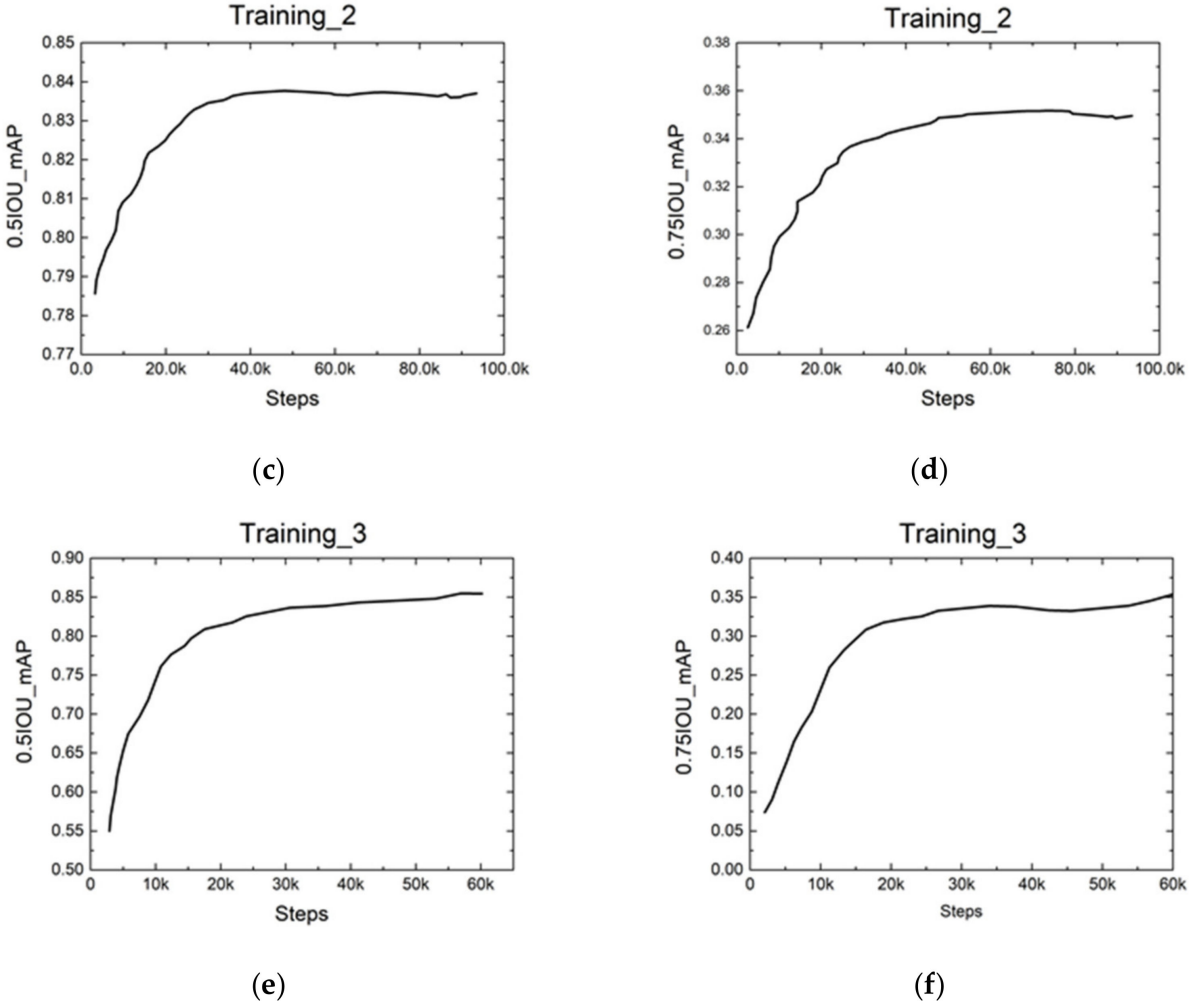
The results of mAP over the training process: (**a**,**b**) the results over the first training step; (**a**) the increase in mAP@0.5IOU over steps; (**b**) the increase in mAP@0.75IOU over steps; (**c**,**d**) the results over the second training step; (**c**) the increase in mAP@0.5IOU over steps; (**d**) the increase in mAP@0.75IOU over steps; (**e**,**f**) the results over the third training step; (**e**) the increase in mAP@0.5IOU over steps; (**f**) the increase in mAP@0.75IOU over steps.

Figure 13 shows the results over the training process. The Intersection of Union (IOU) is defined as the area of intersection divided by the area of union between the predicted bounding box and the ground-truth bounding box. It has been widely used to measure the accuracy of object detection tasks. A value of 0.5 or 0.75 was the threshold we used to judge whether the location of the predicted crack was correct or not. From the results, we could see that the value of mean average precision (mAP) increased quickly at the beginning and slowly stabilized in the end. 

Table 1 summarizes the final results. In the training process, the mAP increased from 0.71 to 0.86 at 0.5IOU and increased from 0.29 to 0.35 at 0.75IOU. It could also validate that the parameters converged to the global optimum by changing the learning rate. 

**Figure 14:**
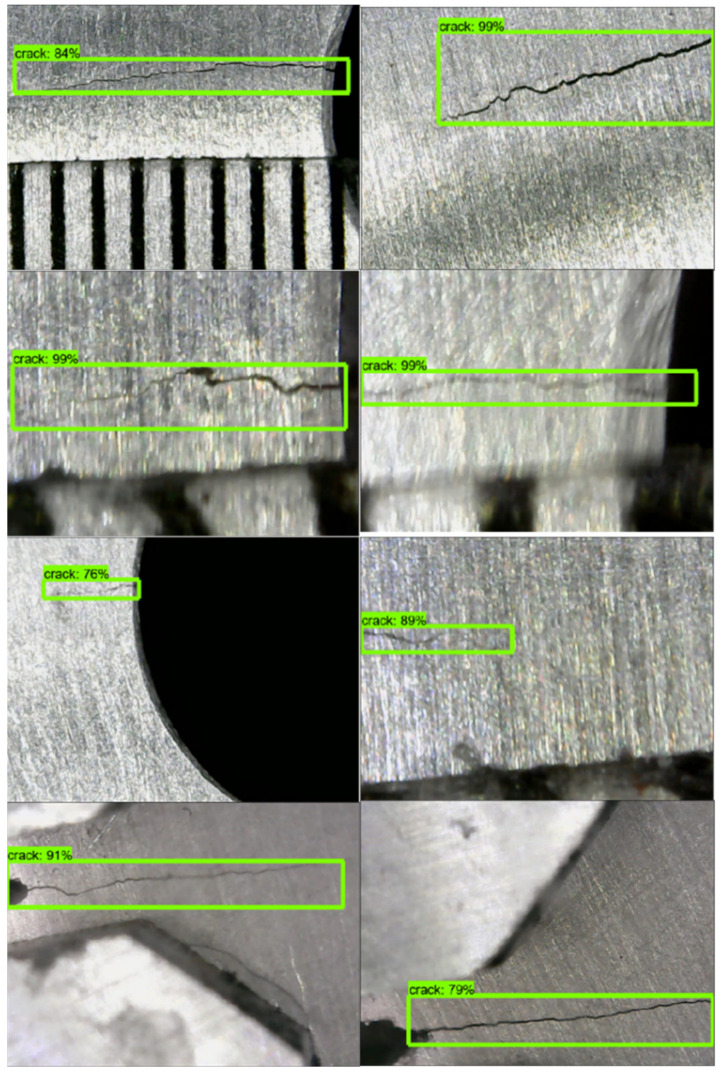
Crack detection results.

After the training, the trained CNN model was applied to other crack images for further validation. As shown in Figure 14, the trained CNN model could detect cracks with high precision not only for large cracks but also for small cracks. It could also detect cracks in different resolution images. At the same time, the method could divide the crack from crack-like noises effectively.

### 3.3. Crack Tip Location Results 

**Figure 15:**
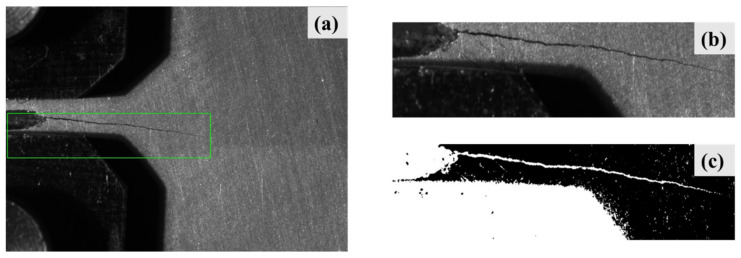
Results after CNN detection and threshold segmentation: (**a**) crack detection results; (**b**) extracted crack region; (**c**) crack region after threshold segmentation.

This section describes the results of the crack tip detection method. Figure 15a shows the results after locating the area of cracks using the trained CNN model. The region of the crack was extracted as a new image, which is shown in Figure 15b. Figure 15c shows the result of a binary image after image segmentation. In this study, the grayscale value *T* was 83. If the pixel gray value was higher than 83, it would be redefined as 0. Otherwise, if the pixel gray value was lower than 83, the number would be redefined as 255. 

**Figure 16:**
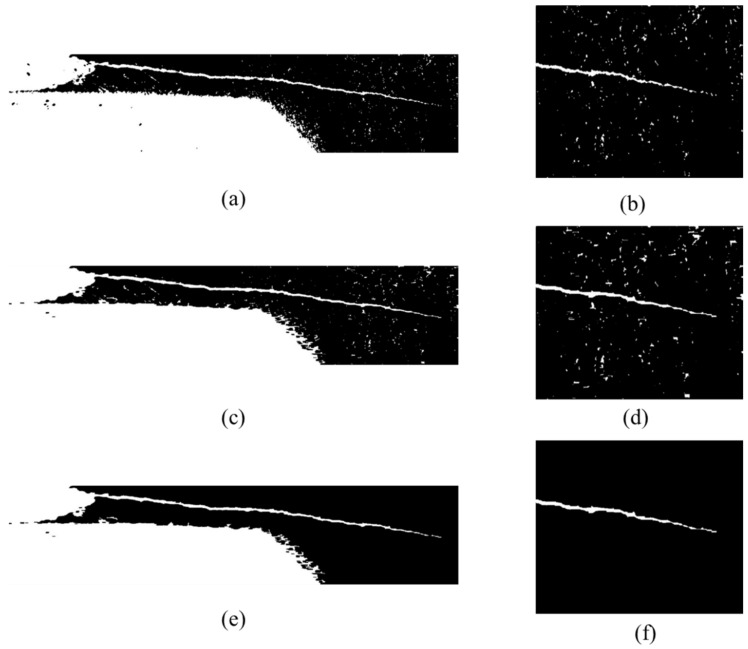
Results after morphological operation and small area removal: (**a**) crack region after threshold segmentation; (**b**) crack tip after threshold segmentation; (**c**) crack region after morphological operation; (**d**) crack tip after morphological operation; (**e**) crack region after small area removal; (**f**) crack tip after small area removal.

As we can see in Figure 16b, the crack tip was disturbed after global segmentation. The crack tip information would be easily missed in the next operation. Before removing the noises in the picture, we connected the crack tip first. Figure 16c shows the results of the crack region after morphological operation. We designed a rectangular structuring element with length of 15 and width of 3, using the element with the closing operation to connect the crack. The crack tip image before connection and after connection can be seen in Figure 16b,d. The different regions of the crack were perfectly connected after the closing operation.

Figure 16e,f show the results of the crack image after moving the noises area. As we can see, the noises could be moved clearly while the number of area thresholds was set as 200. At this point, we could recognize the crack tip as the rightmost point where the greyscale value was 255. 

### 3.4. Accuracy Evaluation Results 

**Figure 17:**
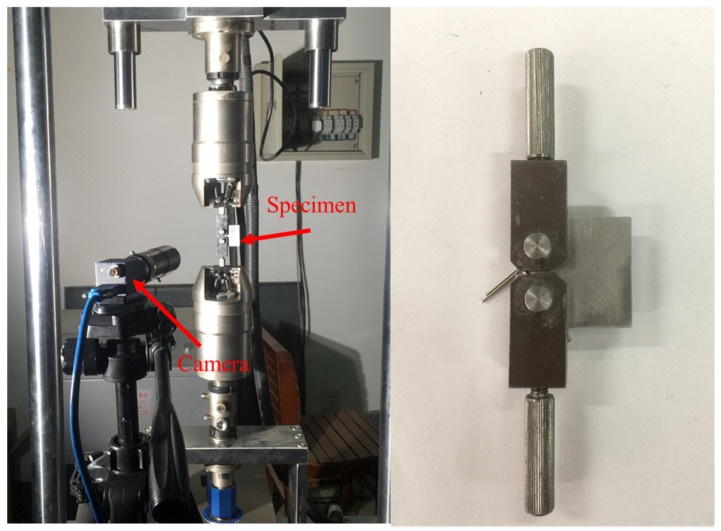
Experimental environment and the CT specimen.

This section shows the results of evaluation for the proposed method in fatigue experiments. The fatigue experimental environment and compact tensile (CT) specimen are shown in Figure 17. A camera was used to take pictures of cracks under different fatigue load cycles. The resolution of the images was 3840 × 2748. The specimen was made of aluminum alloy. 

**Figure 18:**
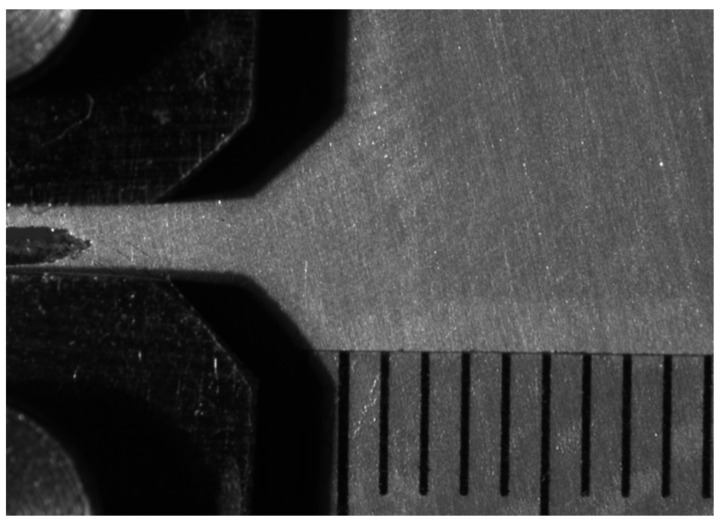
Crack image calibration method.

During a fatigue experiment, the crack length will increase. The crack length reference point detection method was the same as for the detection of the crack tip. The crack length could be calculated as the difference in horizontal value between the crack tip and the reference point. Then, the distance was converted into physical distance from pixel distance. The scale ruler was pasted on the surface of the specimen as shown in Figure 18. Each division on the scale was 1 mm. After calibration, we obtained the result that 1 mm was equal to 219.5 pixels, which meant 1 pixel was equal 4.56 × 10^−3^ mm.

**Figure 19:**
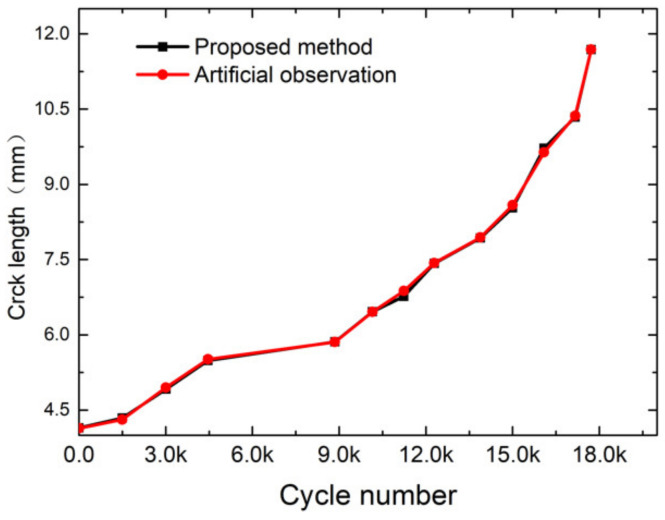
Crack length measurement results over fatigue experiments.

**Figure 20:**
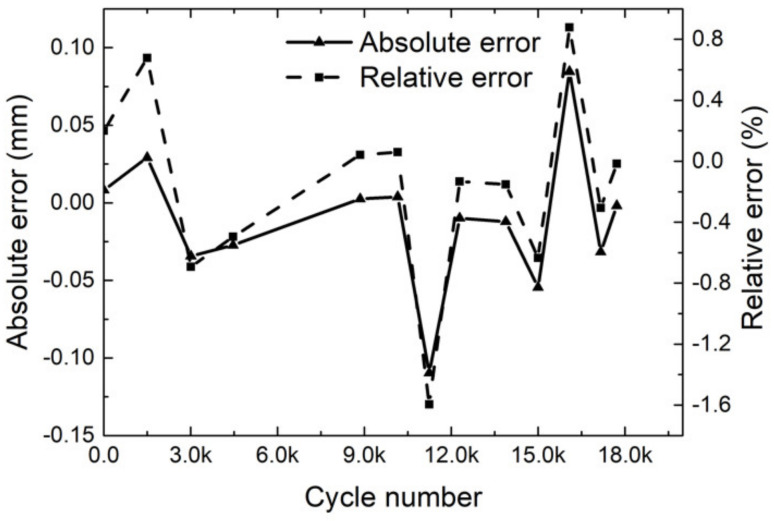
The absolute error and relative error of crack length.

**Figure 21:**
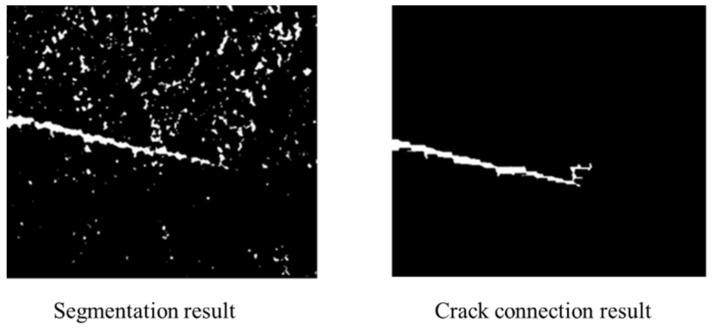
Image details of the reason for errors.

Figure 19 illustrates the crack length results of the proposed method and the results of human inspection. We used a measuring magnifier to check the crack length and compare the results with the proposed method. The crack length increased with the fatigue cycles. Figure 20 illustrates the absolute error and relative error at different stages. Comparing crack length results measured by the proposed method with the human inspection method, the maximum absolute error was less than 0.15 mm, and the maximum relative error was less than 2%. Figure 21 explains why the errors existed. The morphology operation not only connected the different crack areas, but also the noises and the crack. As a result, the error of crack length calculated was larger. According to the comparison results, the crack length could be measured effectively and accurately by the proposed method in this paper. 

## 4. Conclusions

In this paper, a non-contact framework for the fatigue crack length measurement is presented through the study of a robust crack area location method and crack tip detection method. A crack image dataset was established for the training of CNN. The trained CNN could locate the crack accurately with the inference of crack-like edges, which enhanced the robustness of the proposed method. Then, a new algorithm for crack tip location was presented. First, the extracted crack image was converted into a binary image. Next, the morphological operation was used to connect the disturbed crack. The noises in the images were removed by setting an area threshold. Finally, the crack tip could be easily detected. The performance of the proposed method was experimentally validated through a CT specimen fatigue test. The results demonstrated that the proposed method could be used to measure crack length effectively and accurately. The method proposed in this paper could be used to identify the location of cracks from images and monitor crack length during fatigue experiments. Its application will improve the study of fracture mechanics. This work established a new framework for measuring crack length by locating the crack tip. A limitation of this study was that the image segmentation threshold was manually selected to keep the information about the crack tip. An automatic image segmentation method needs to be developed in the future. Another further study will focus on implementing this method on mobile platforms for automated crack inspection. Furthermore, crack width is an essential parameter for the serviceability of reinforced concrete construction. The proposed method will need to be modified to meet crack width measurement requirements.

## Figures and Tables

**Table 1:** Crack detection precision.

Result	First Step	Second Step	Third Step
mAP (0.5IOU)	0.71	0.84	0.86
mAP (0.75IOU)	0.29	0.34	0.35

## Data Availability

Data sharing is not applicable to this article.
